# Mapping and phylogeny of *Biomphalaria* snail in the Adamawa Region of Cameroon: A step towards vector control and schistosomiasis elimination

**DOI:** 10.1371/journal.pntd.0013265

**Published:** 2025-06-27

**Authors:** Bakari Fadilatou Foule, Mureille C. Tchami Mbagnia, Romuald I. Kamwa Ngassam, Alvine C. Kengne-Fokam, François S. Ngambia Freitas, Daniel Nguiffo Nguete, Hugues C. Nana Djeunga, Charles S. Wondji, Tito T. Melachio Tanekou, Flobert Njiokou

**Affiliations:** 1 Laboratory of Parasitology and Ecology, Department of Animal Biology and Physiology, Faculty of Science, University of Yaoundé I, Yaoundé, Cameroon; 2 Department of Biological Sciences, Faculty of Science, University of Maroua, Maroua, Cameroon; 3 Centre for Research in Infectious Diseases (CRID), Yaoundé, Cameroon; 4 Translational Research and Development Foundation (TREND Foundation), Yaoundé, Cameroon; 5 Department of Vector Biology, Liverpool School of Tropical Medicine, Liverpool, United Kingdom; 6 Department of Microbiology and Parasitology, Faculty of Science, University of Bamenda, Bamenda, Cameroon; University of Queensland & CSIRO Biosecurity Flagship, AUSTRALIA

## Abstract

**Background:**

Schistosomiasis is the world’s second-most important parasitic disease affecting humans. Among the two main forms of the disease, intestinal schistosomiasis due to *Schistosoma mansoni* is predominant in Cameroon, where its intermediate host *Biomphalaria* spp. is widely distributed, particularly in the Adamawa plateau. As a prerequisite to targeted vector control for effective elimination of intestinal schistosomiasis infection, we mapped the geographical distribution of *Biomphalaria* snails in the Adamawa Region.

**Methodology/Principal findings:**

A total of 43 human-water contact sites were visited across the Adamawa Region for snail collection. Snail species were identified morphologically and with PCR-RFLP technique at the ITS2 rDNA region using the *Hpa*II restriction enzyme. The genus *Biomphalaria* was identified at 13 sites (30.2%), four sites (9,3%) harboured *Gyraulus* species firstly identified as *Biomphalaria* with shell morphology, and 22 sites (51,16%) were free from any snail species. Two *Biomphalaria* species were identified, *Biomphalaria pfeifferi* in 12 water contact sites, and *Biomphalaria camerunensis* was found only in one site (Djalingo, Vina Division), and it was the first report of this species in the Northern Cameroon (above the 6° latitude North). Morphologic identification was supported by PCR-RFLP results and sequencing revealed three haplotypes for *Biomphalaria pfeifferi* and one haplotype for *Biomphalaria camerunensis*. The studied populations were stable according to neutrality tests (Tajima’s *D* and Fu *Fs*) and no signal of gene flow was observed between them.

**Conclusions/Significance:**

This study confirmed the presence of *Biomphalaria pfeifferi* in the Adamawa Region and reported for the first time *B. camerunensis* above 6° of Latitude North, thus deserving further monitoring to assess its current distribution in Cameroon.

## Author summary

The transmission of schistosomiasis depends on the distribution of specific freshwater snails that act as intermediate hosts for the parasite’s larval form. The World Health Organization recently recognised that efforts to interrupt schistosomiasis transmission through mass drug administration solely have not been sufficient in some regions to eliminate the infection and encouraged the control of these intermediate hosts as a complementary intervention. In this study, we mapped the distribution of *Biomphalaria* snails throughout the Adamawa Region of Cameroon and assessed the phylogenetical relationship between different populations as a prerequisite to snail control. *Biomphalaria* snails were found in four of the five Divisions of the Adamawa Region. In addition to *B. pfeifferi* that was the only species reported in previous studies, *B. camerunensis* was identified in the region for the first time. Genetic analysis revealed a slight intraspecific variation in *B. pfeifferi* and, that snail populations are stable on the field.

## Introduction

Schistosomiasis is an important snail-borne parasitic disease prevailing in tropical and subtropical regions. Although the disease is both treatable and preventable, it remains a major concern in several low and middle-income countries with more than 90% of cases found in sub-Saharan Africa [1]. About 200 million people are infected worldwide, and over 700 million people are at risk of infection [2,3]. In Cameroon, schistosomiasis is reported to have been endemic for more than three decades, with a nationwide distribution [4,5]. Two million cases were reported in 2009, with around 5 million people at risk of infection [6]. The intestinal schistosomiasis is the most largely distributed form [4] affecting the entire northern part of the country, from the Chad basin to the Adamawa Region [4,5], where a high prevalence is recorded in several foci [6].

Water contact behaviour is the main factor driving the spread of schistosomiasis. Indeed, people come into contact with water for several reasons, either directly at the water sources or by using water previously collected [7] especially for personal care, domestical and recreational purposes [2,8,9]. This contact increases their risk of infection through skin penetration by the cercariae, the larval stage of the parasite that are shed in water by *Biomphalaria* intermediate hosts. Despite years of annual mass drug administration (MDA) with praziquantel [10] and community behavioural change programs [11], the Adamawa Region remains hyperendemic for schistosomiasis. The transmission of schistosomiasis depends on the distribution of specific freshwater snails that act as intermediate hosts for schistosomes [12,13]. In this line, *Biomphalaria pfeifferi* has been described as the main intermediate snail host for *S. mansoni* in the Adamawa Region of Cameroon [4,5]. Because of job seeking, instability related to wars and terrorism, there is an important population movement/migration from different Regions of Cameroon or from neighbouring countries (Nigeria, Central African Republic and Chad) - where intestinal schistosomiasis is highly endemic - to the Adamawa Region. These population movements can exacerbate and perpetuate the circulation of *Schistosoma mansoni* in the region, leading to the occurrence of new transmission sites, particularly where intermediate host snails are already present [14–16]. Although several malacological surveys have been carried out nationwide, updated data on the distribution and abundance of *Biomphalaria* snails in the Adamawa Region are very scanty. Given the persistence of schistosomiasis burden despite the uninterrupted routine interventions (deworming campaigns, health education and water sanitation and hygiene (WASH) programmes), the World Health Organisation (WHO) highlighted the need of snail control for global schistosomiasis control and elimination [17–22]. Mapping snail populations therefore appears as pre-requisite for the delineation and categorisation of transmission risk areas for effective snail control and monitoring programmes [23].

In this study, the main hypothesis formulated was that physicochemical parameters could shape the distribution of *Biomphalaria* snail species in the Adamawa Region of Cameroon. We therefore aimed to map the geographical distribution of *Biomphalaria* spp. in the Adamawa Region, determine the potential influence of physicochemical parameters in their distribution and assess the phylogenetical relationship between different populations, to contribute to the development of appropriate preventive measures like effective snail control programmes to foster elimination of schistosomiasis.

## Methods

### Study area

The Adamawa Region is a mountainous area at the transition between the forested areas of the south and the savannas of the north of Cameroon, mainly consisting of highlands with average altitudes of 1,100 metres [24]. The Region is located at the edge of the western volcanic chain and borders Nigeria at the west and the Central African Republic at the east. The tropical-Sudanese climate in this region is characterised by two seasons: the dry season, from November to mid-April followed by the rainy season from mid-April to late October. The mean rainfall ranges between 900 and 1,500 mm, with more significant declines noticed in the north [25]. It is worth mentioning that the Adamawa Region is considered as the Cameroon’s “water tower” due to its important hydrographical network with several river sources. Livestock farming, agriculture and commerce are the main activities in the Region.

### Collection sites and snail surveys

All human-water contact sites accessed by local communities for various purposes were surveyed in all the five Divisions (Djerem, Faro et Deo, Mayo Banyo, Mbere and Vina), that is 21 Sub-Divisions of the Adamawa Region, from December 2021 to January 2022 (S1 Table). These sites were identified following information collected from community leaders and villagers and were georeferenced using a handheld global positioning system (GPS) (eTrex, Garmin Ltd, Southampton, UK).

Snails were collected with gloved hands from the mud, in areas with shallow water bodies, on dead leaves or solid surfaces, or by scooping the water body floor with handheld snail scoop for 30 min per site [26]. All snails collected were classified by genus based on shell morphology, counted and recorded in the sampling book [7]. Only schistosome intermediate hosts were individually fixed in labelled tubes containing 95% ethanol. Samples were taken back to the Parasitology and Ecology Laboratory of the University of Yaoundé I, Cameroon and stored at -20°C for further molecular studies [27].

Environmental factors (substrate, riverbank characteristics, human activities and presence of animals) and physicochemical parameters of water (Turbidity, water temperature, pH, electrical conductivity, total dissolved solid, salinity and redox potential) were recorded onsite before snail sampling. Physicochemical parameters of water were measured using a Noyafa NF- C600 Digital 7 in 1 multiparameter handheld water quality tester (Shenzhen, China).

### Snail identification

The species of all snails from the genus *Biomphalaria* were identified using conchological criteria such as shell height and width, aperture height and width and body whorl height, using the key defined by Brown [28], and 15 individuals from each site were randomly selected for molecular analyses. For each selected individual, DNA was extracted using Cethyl Trimethyl Ammonium Bromide (CTAB) extraction method as previously described [27] and stored at -20°C until PCR amplification. The ITS2 region of the genome ribosomal DNA was amplified using ITS2F (5′-CGTCCGTCTGAGGGTCGGTTTGC-3′) [29] and ETTS1 (5′-TGCTTAAGTTCAGCGGGT-3′) primers [30]. All PCR reactions were performed in a final volume of 40 μL containing 4 μL of extracted DNA, 4 μL of PCR buffer (10X) (Kapa Biosystems, Cape Town, South Africa), 1.6 μL of each of forward and reverse primers (10 μM), 0.8 μL of dNTPs mixture (10 mM), 0.12 μL of Taq DNA polymerase (5 U/μL) and 27.88 μL of distilled water. The PCR cycling conditions included an initial denaturation at 95°C for 3 min 30 sec, followed by 35 cycles made up of a denaturation step at 95°C for 30 sec, a primers’ annealing step at 60°C for 30 sec, and an extension step at 72°C for 30 sec, followed by a final extension at 72°C for 10 min. Five microliters from all PCR products were resolved on 2% agarose gel electrophoresis containing ethidium bromide and visualised under UV light [27] to ensure good amplification. Then, 2 μL of amplified DNA were digested at 37°C for two hours using *Hpa*II restriction enzyme, in a 25 μL final reaction mixture volume containing 1 μL of restriction enzyme, 5 μL of the manufacturer’s buffer and 17 μL of distilled water [27]. Digested products were resolved on 8% polyacrylamide gel containing ethidium bromide and visualised under UV light.

### Sequencing and genetic diversity of *Biomphalaria* spp

Ten microliters of amplified DNA samples were purified using the enzymatic PCR clean up method with Exonuclease I (ExoI) and shrimp alkaline phosphatase (SAP) (New England Biolabs, Boston, MA, USA) as recommended by the manufacturer and sent for Sanger sequencing in both directions in commercial companies, i.e., Microsynth Seqlab GmbH (Göttingen, Germany) and Inqaba Biotec (Ibadan, Nigeria).

For each sample, forward and reverse sequences received were edited using Chromas software V2.6.6 (https://technelysium.com.au/wp/chromas/) and aligned together to obtain consensus sequences using the software BioEdit V7.1.9 [31]. Consensus sequences were nucleotide blasted (Blastn) in GenBank nucleotide (nr) database (https://blast.ncbi.nlm.nih.gov/) for comparison with archived sequences, and species attributed to each sample was the one presenting high similarity (identity >  98%. E-value <<< 0.001) with the query sequence submitted. The DNA sequences generated are deposited in GenBank (accession numbers: PQ783664 to PQ783677 for *Biomphalaria* ITS2 fragments and PQ783678 for *Gyraulus* ITS2 fragment). Consensus sequences were aligned using the multiple sequence alignment tool (ClustalW) algorithm with BioEdit software for phylogenetics analyses [31].

### Statistical analyses, genetic variability and distribution mapping of *Biomphalaria* spp

Statistical analyses were undertaken using IBM SPSS Statistics version 23 (SPSS Inc, Chicago, IL, USA) and R Statistical Software (v4.1.2; R Core Team 2021), with 5% significance threshold. The non-parametric Kruskal-Wallis test was used to compare the mean number of snails collected according to the different categorical variables of the water physicochemical parameters (water body type categorized into spring, river and pond, water turbidity categorized into high, medium, and low, and presence of mud or sand). Multiple linear regression was performed to assess the relationship between snail abundance (dependent variable) and continuous variables of the physicochemical water parameters (pH, altitude, temperature, conductivity and total dissolved solids), the latter being considered as independent or explanatory variables (S2 Table). This regression was done after checking independence of explanatory variables, with a multicollinearity test performed in R software.

The genetic variability of each species [32] was assessed using DNASP5.10.01 [33] with estimation of the number of polymorphic sites (S), Haplotype (gene) diversity (Hd), nucleotide diversity (π) [34], Fu’s *Fs* statistics and Tajima’s *D* [35]. The genetic differentiation between populations was estimated with the fixation index (*Fst*) and a Phylogenetic tree was constructed using pairwise distances calculated with the maximum likelihood method based on the Jukes-Cantor model [36], implemented in the software MEGA11 [37]. A haplotype network was constructed with different haplotypes using TCS v1.21 software to better visualise phylogenetic relationships between populations sampled [38] and mutational steps diagram was generated using DNASP5.10.01 and MEGA11.

The software ArcGIS version 10.8 (ESRI Inc., Redlands, CA, USA) was used to map the distribution and abundance of snails collected in the sampling sites. The ESRI basemap and GADM shapefiles (https://gadm.org/maps/CMR_1.html) used for this purpose are freely available for research as stated in the terms of use (available here and here respectively). Three main layers (roads, vegetation cover and rivers/streams, the latest representing potential snail breeding sites) were used to create the map.

## Results

### Occurrence of freshwater snails

A total of 43 water contact sites were investigated and planorbid snails were found in 21 sampling sites (48.8%), where 1,670 snails were collected. Of these, 1,092 (65.4%) were identified as the *Biomphalaria* genus using morphological criteria. These specimens were collected alone in seven sites, while they were found in sympatry in eight sites with other planorbids like *Bulinus* spp, the *Schistosoma haematobium/ Schistosoma guineensis* intermediate snail hosts. The colonized sampling sites were distributed in four of the five Adamawa’s Region Divisions (Table 1). Twenty-two water contact sites were found to be free from any snail species at the time of collection.

**Table 1:** Different collection sites with *Biomphalaria* spp. snails.

Division	Subdivision	Village	Collection site	Site type	No. snails collected
Djerem	Ngaoundal	Ngaoundal	Mbilo	River	64
Faro et Deo	Mayo Baleo	Mayo baleo	Dokdoure	River	36
		Mayo Baleo	Mayo Baleo*	River	56
		Alme	Mayo Alti**	River	8
		Alme	Dilecty	Stream	90
	Tignere	Woulde	Mayo Petel**	Stream	48
Mayo - Banyo	Banyo	Banyo	Mayanka	River	26
		Banyo	Pendeng	River	218
Vina	Mbe	Kana Petel	Mayo Mvôo*	River	46
		Selou-Semba	Mayo Tchoro	River	108
	Nganha	Berem	Maporo	River	86
	Ngaoundere 1	Beka Hossere	Beka Hossere	Stream	136
		Beka Hossere	Mardock	River	56
	Ngaoundere 2	Djalingo	Djalingo	River	8
	Ngaoundere 3	Tchabbal	Tchabbal	Lake	106

*All specimens latter identified as *Gyraulus* sp with the molecular assay; **Biomphalaria sp. latter found in sympatry with *Gyraulus* sp.

### Features of the sampling sites and correlation with snail abundance

A great variation was observed in the physicochemical parameters of the different sites (S2 Table). The mean temperature was 23.8 °C (SD: 4.25°C) ranging from 7.9°C to 31.7°C. The mean pH was 5.71 (SD: 1,25), ranging from 4.73 to 11.0. The conductivity ranged from 8 µs/cm to 123 µs/cm and total dissolved solids (TDS) values ranged from 4 PPM to 131 PPM, with a mean of 35 PPM (SD: 32.3) and a median of 23 PPM (25%-75% IQR: 14–54). A total of 83% of sampling sites were characterised by the presence of sheep/goats, 63% by the presence of dogs, 88% by the presence of waterbirds, and 36% by the presence of donkeys. A positive association was found between the planorbid snail abundance and electrical conductivity/or total dissolved solids (these two explanatory parameters were highly and positively correlated, as shown by the collinearity test (S1 Fig; r > 0.99, p < 0.0001)). Anyway, the association test showed a significant increase in planorbid snail abundance with the increase of site TDS or conductivity (p-values of 0.016 and 0.02 respectively). However, no significant correlation was observed between the snail abundance and turbidity, temperature, pH, salinity, redox potential as well as environmental physical parameters (all p-values > 0.05).

### Conchological identification of *Biomphalaria* species

**Fig 1:**
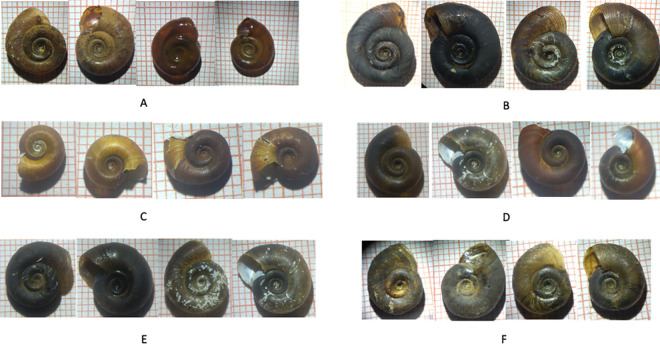
Shell morphology and size in *Biomphalaria* populations sampled. (A) represents *Biomphalaria camerunensis* from Djalingo, and B, C, D, E, and F represent *Biomphalaria pfeifferi* from Mayo Tchoro, Mayanka, Mbiolo, Mayo Mvô’o and Beka Hossere sites, respectively.

Despite different shell sizes and shapes observed in several sites (Fig 1), two *Biomphalaria* species were identified based on conchological criteria: *Biomphalaria camerunensis*, with shell diameters ranging from 11 to 13 mm, that was found in Djalingo (Fig 1A), whereas *Biomphalaria pfeifferi*, with shell diameter ranging from 7 to 18 mm, was found in 14 localities. Snails from Mayo Tchoro identified as *B. pfeifferi* (Fig 1B) appeared very similar morphologically to *B. camerunensis* specimens than to the other *B. pfeifferi*.

### Molecular identification of *Biomphalaria* species

**Fig 2:**
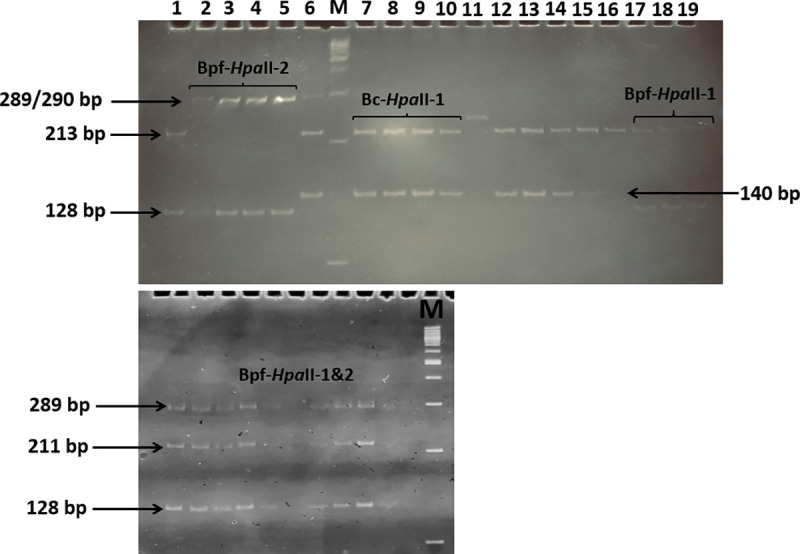
Polyacrylamide gels showing the different profiles identifying *Biomphalaria pfeifferi* (Bpf-*Hpa*II-1, Bpf-*Hpa*II-2 and the hybrid Bpf-*Hpa*II-1&2) and *Biomphalaria camerunensis* (Bc-*Hpa*II-1) after digestion of ITS2 amplified fragments with the restriction enzyme *Hpa*II; M: molecular weight marker (100 bp lanes).

**Fig 3:**
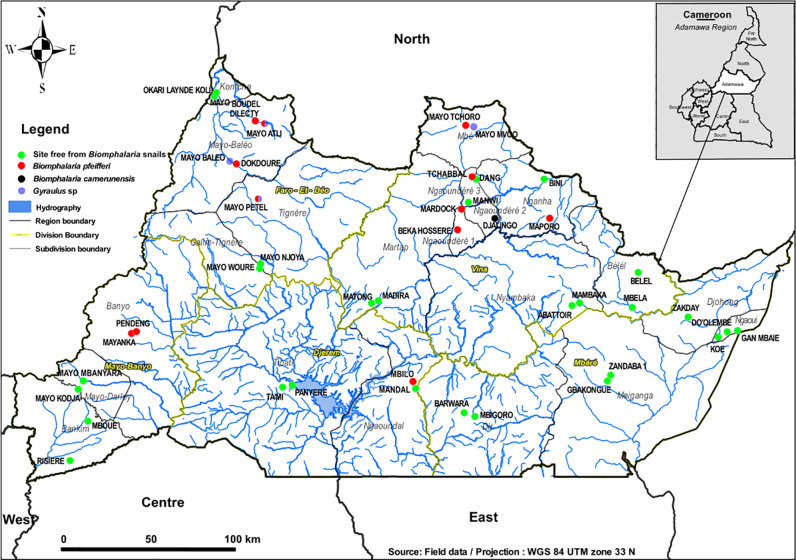
Map showing the geographical distribution of *Biomphalaria* species in the different collection sites.

The amplification of ITS2 fragments of 193 snails (around 15 per site) morphologically identified as *Biomphalaria* sp. showed fragments of ~490 bp as expected for these species, for 156 individuals. The digestion of these PCR products with the restriction enzyme *Hpa*II revealed the profiles of *B. camerunensis* and *B. pfeifferi* (Fig 2), confirming their identification based on shell morphology. Indeed, the *B. camerunensis* profile Bc-*Hpa*II-1 (with ~212 bp and ~139 bp bands) was identified only in Djalingo site, the *B. pfeifferi* profile Bpf-*Hpa*II-1 (with ~211 bp and ~128 bp bands) was identified in Manyaka in Banyo, while the profile Bpf-*Hpa*II-2 (with ~289 bp and ~128 bp bands) was identified in nine other sites distributed across the study area (Fig 3). Moreover, a new profile made of the combination (hybridization) of the two previous ones (289 bp, 211 bp and 128 bp) was observed in all individuals screened in two sites, Mbilo and Dilecty (15 and 12 individuals respectively), and in two individuals from Pendeng (out of 15). However, 100% of individuals screened from Mayo Baleo (14) and Karna Petel (15), 8,33% (2/24) in Alme and 46,15% (6/13) in Woulde that were morphologically similar to *B. pfeifferi* displayed ITS2 fragments of ~540 bp. The sequencing and alignment of five of these PCR products with reference sequences in Genbank using the blastn algorithm revealed that the most related sequences were those of *Gyraulus* sp (93% identity, E-value = 0).

The map was designed using the ESRI satellite basemap (https://www.arcgis.com/home/webmap/viewer.html?url= https%3A%2F%2Fserver.arcgisonline.com%2Farcgis%2Frest%2Fservices%2FWorld_Imagery%2FMapServer&source=sd) and GADM shapefiles (https://gadm.org/maps/CMR_1.html) for boundaries which are freely available for research as stated in the terms of use (available here and here respectively).

### Genetic diversity in *Biomphalaria* species

From the seven *Biomphalaria pfeifferi* populations analysed with the sequencing of their ITS2 fragments, a total of three haplotypes (alleles) were detected with PCR-RFLP, corresponding to the profiles Bpf-*Hpa*II-1 (H1), Bpf-*Hpa*II-2 (H2) and the hybrid Bpf-*Hpa*II-1&2 (H3). All individuals screened in Mayanka were homozygotes with the first haplotype, all specimens from Mayo Petel, Mardock, and Maporo were homozygotes with the second haplotype, and all specimens from Mbilo and Dilecty were hybrid genotypes with both haplotypes. In Pendeng, 11 (84,6%) snails were homozygous with the second haplotype, while 2 (15,38%) were heterozygotes. Only one allele corresponding to the PCR-RFLP profile Bc-*Hpa*II-1 was detected for *B. camerunensis* collected in Djalingo. The overall haplotype (Hd) and nucleotide diversities (π) of *B. pfeifferi* samples were 0.500 and 0.006, respectively. Based on neutrality tests, *Biomphalaria* populations in different sites showed a deficiency in polymorphism as indicated by an overall positive but not significant Tajima’s *D* (1.140), and a positive and significant Fu’s Fs statistic (FS = 8.264, P < 0.001).

### Phylogenetic analyses

**Fig 4:**
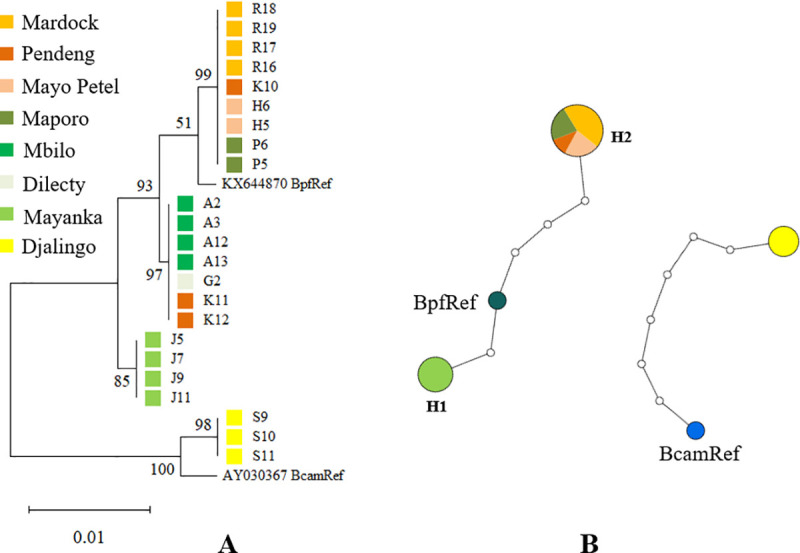
Maximum likelihood phylogenetic tree (A) and haplotype network (B) constructed with ITS2 sequences for *Biomphalaria pfeifferi* and *Biomphalaria camerunensis.* The two alleles were concatenated to capture the hybrid variant in the phylogenetic tree; sizes of the circles are proportional to the haplotype frequencies in the haplotype network and hybrids could not be included here. KX644870: reference ITS2 accession number of *B. pfeifferi*. AY030367: reference ITS2 accession number of *B. camerunensis*.

The phylogenetic tree constructed using ITS2 gene fragment showed that all the three haplotypes of *B. pfeifferi* (H1, H2 and H3), even though present on different sub-clusters, were closely related compared to *B. camerunensis* that formed a second cluster with a 100% bootstrap (Fig 4A). The haplotype network built showed that the haplotype (H2) was the most represented while H1 was found only in Mayanka population (Fig 4B).

**Fig 5:**
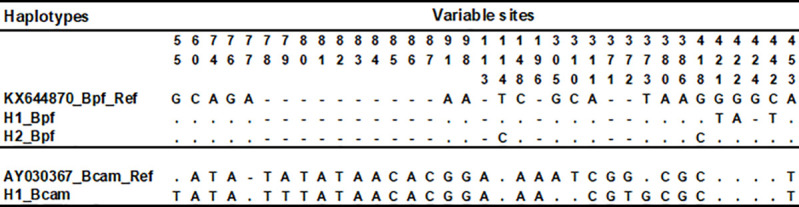
Variable sites observed in the two haplotypes recorded for *B. pfeifferi* (Bpf) and *B. camerunensis* (Bcam) using the reference sequences with accession numbers KX644870 and AY030367 for the two species respectively.

The haplotypes H1 and H2 differed from the *B. pfeifferi* reference sequences of individuals collected in the Centre Region of Cameroon with four and two mutational steps respectively, while *B. camerunensis* haplotype from Djalingo differed from Cameroon *Biomphalaria camerunensis* reference sequence with seven mutational steps (Fig 5).

## Discussion

Effective management of snail populations can significantly reduce transmission rates and contribute to the elimination of schistosomiasis, which control had long relied on MDA only [39]. As prerequisite to effective snail control, identifying and mapping the species present in endemic areas is essential for optimal allocation of control means [1,40]. In the present study, we mapped the distribution of the *Biomphalaria* snails, intermediate hosts of *Schistosoma mansoni* in the Adamawa Region of Cameroon, to support upcoming snail control programmes.

### Snail distribution

Snail distribution was found to be very heterogenous between sampling sites, indicative of the complexity of the distribution of schistosomes intermediate hosts in Cameroon as previously reported [4,41]. This can be explained by the complex set of factors including climate, habitats, hydrological conditions and vegetation, that influence the distribution of these organisms [4]. Among the physicochemical variables measured in this study, electrical conductivity and total dissolved solids were positively associated with snail occurrence as previously described in Omo-Gibe River basin, Southwest Ethiopia [42]. It is not well understood how these physicochemical parameters affect the snail biology and distribution since they vary considerably according to seasons as reported in previous studies in Uganda [43,44]. However, high levels of electrical conductivity may be associated to the presence of ions such as calcium, silicium and magnesium deriving from dissolved solids (thus the positive correlation between both parameters), and these ions are needed for shell development and therefore, favour snail occurrence and abundance, as previously reported by Allouko et al. [45]. Furthermore, some parameters like water temperature and concentration of dissolved oxygen were found associated to snail presence and abundance in some settings, while they were not associated in others [46]. Further research is still needed to provide more knowledge on how environmental and biotic factors influence freshwater snail intermediate hosts distribution that could help predicting their presence in different settings.

### *Biomphalaria* species identification

Based on conchological criteria, 14 snail populations were identified as *Biomphalaria pfeifferi*, while one population (Djalingo) was identified as *B. camerunensis*, what was strange since it was accepted that *B. camerunensis* is not present above the 6° of latitude north, lower limit of the Adamawa Region [27,41]. Molecular analyses confirmed the presence of *B. camerunensis* in Djalingo, that displayed the PCR-RFLP common profile Bc-*Hpa*II-1 previously described for the species in many other sites of the Southern Regions of Cameroon [47]. This is the first report of *B. camerunensis* above the 6° latitude North in Cameroon, suggesting either previous misidentification of this species, or their recent colonisation in new areas. Misidentification can occur particularly in *Biomphalaria* species due to overlapping morphological traits among different species as reported in previous studies [48–50]. In this study, *B. pfeifferi* individuals sampled in Mayo Tchoro were very similar to *B. camerunensis*, with around six slowly increasing whorls and shells of bigger sizes (up to 18 mm), while the umbilicus was smaller and typical of *B. pfeifferi.* The molecular assay later confirmed these specimens as belonging to *B. pfeifferi*, as well as the species of the other populations sampled. Moreover, all the snails from Mayo Mvô’o and Mayo Baleo sites plus part of those collected in Mayo Alti and Mayo Petel, initially identified as *B. pfeifferi* with morphologic characteristics were later found to be *Gyraulus* sp with the molecular assay. Although criteria like the height of shell allow to distinguish *Gyraulus* sp (less than 2mm) from *Biomphalaria* spp. (up to 6 mm) at the adult stage [49], it is more difficult to differentiate these specimens at a juvenile stage. In our study, due to the restriction from the funding body of using the funds after January, the sampling was made from December to January, when most of the populations were still being established and therefore still at a juvenile stage. However, the best-known sampling period is February to April, where most populations have reached the adult stage. This was a limitation in our study, but the result reinforces the importance of using molecular tools like the simple PCR-RFLP previously developed for the precise identification of these snails [47,50]. Apart from the previous misidentification of *B. camerunensis*, there is a possibility of its recent expansion in the northern part of the country due to climate change or population/cattle migrations through the area. Climate change can alter freshwater ecosystems, making them more suitable for certain snail species to thrive and expand their range [2]. Additionally, human activities, including population movements and cattle grazing with instability in the northern Cameroon, could have created new water bodies or modified existing ones, thereby providing opportunities for snails to establish themselves in previously unsuitable environments. Since *B. camerunensis* is known for its great efficiency to transmit and disseminate *S. mansoni* [14], its presence in the Adamawa Region can exacerbate the risk of transmission of *S. mansoni*. The interplay between these factors highlights the need for ongoing monitoring and mapping of snail populations to better understand their distribution for sustainable snail control programmes [39,40].

### Genetic diversity and phylogeny

Low genetic diversity (haplotype and nucleotide diversities) was recorded in the studied *Biomphalaria* populations, as previously observed in populations collected in the southern Cameroon [47] and assessed at the same ITS2 locus or with allozymes [51]. This low polymorphism observed could be due to the ITS2 marker used, which was reported less polymorphic than the other regions used for assessing the genetic polymorphism in *Biomphalaria* snails [52]. However, this result still reflects the expected trend in freshwater snails in general, whose biotopes are most of the time temporary, and where they experience population crashes and renewal from a very few numbers of individuals [28]. This observation is also supported by the Tajima’s *D* and Fu’s *Fs* neutrality tests whose values translated a deficit in number of alleles and polymorphism compared to the theoretical expected. The low genetic diversity in *Biomphalaria* snails may be an important factor limiting their adaptability and plasticity to environmental changes and stressors, and their populations may therefore be more vulnerable to control interventions that alter their habitat or introduce biological agents. Additionally, our results show an inequal repartition of the two ITS PCR-RFLP profiles in the Adamawa Region. Indeed, the profile Bpf-*Hpa*II-1 previously identified in *B. pfeifferi* from far North Region was only identified in Mayanka, while the Profile Bpf-*Hpa*II-2, previously identified in the southern Cameroon populations Region was the commonly distributed profile. This result reflects previous findings using allozymes, that showed an inequal distribution of alleles in the different populations [51]. Moreover, each of the two profiles was found alone where present, and surprisingly, the hybrid profile was also found alone in two sites where it was identified. Although the presence of homozygous profiles could be explained by the fragmented habitats of these snails and lack of geneflow and predominance of selfing as previously reported in *Biomphalaria* populations [53–56], the presence of the hybrid profile alone in two sampling sites remained a mystery that can further be explored by the study of self and cross fertilization experiments with individuals from those populations as described by Njiokou et al. [57]. However, the collection of two heterozygotes snails in Pendeng along with 11 homozygotes could be an indication of the fact that the sampling efforts was not sufficient to find all profiles present in the different sites.

## Conclusions

The study underscores the importance for mapping the snail populations of the Adamawa Region of Cameroon, as prerequisite for effective management of this intermediate host that can foster the fight against schistosomiasis. This study revealed the heterogeneous distribution of different genotypes of *B. pfeifferi* and *B. camerunensis* in the Adamawa Region. The identification of *B. camerunensis* above 6° of latitude north marks a significant shift in the current knowledge of its geographical range and potential impact on disease transmission. These results further highlight the need for longitudinal monitoring of these snail populations to better understand the factors shaping the genotypes distribution, in order to adapt and optimize control measures.

## Supporting information

S1 Table Sites screened for snail collection in the Adamawa Region of Cameroon. (DOCX)

S2 Table Physicochemical characteristics and snail occurrence in different sites of the Adamawa Region of Cameroon. (XLSX)

S1 Fig Multicollinearity correlation values between each pair of water physicochemical parameters used to explain planorbid snail abundance. (TIF)
