# Supplementary material for: Mapping and phylogeny of Biomphalaria snail in the Adamawa Region of Cameroon: A step towards vector control and schistosomiasis elimination
Source: PLoS Negl Trop Dis. 2025 Jun 27;19(6):e0013265. doi: 10.1371/journal.pntd.0013265 (PMC12244607; doi:10.1371/journal.pntd.0013265)
Supplement: S1 Table — (DOCX) [file pntd.0013265.s001.docx]

**Supplementary Table S1.** Sites screened for snail collection in the Adamawa Region of Cameroon

| **Division** | **Subdivision** | **Village** | **Collection site** | **Collection date** | **Site type** | **Latitude (N)** | **Longitude (E)** |
| --- | --- | --- | --- | --- | --- | --- | --- |
| Djerem | Ngaoundal | Ngaoundal | Mandal | 20/12/2021 | River | 06°27'25.3" | 013°16'44.8" |
|  |  | Ngaoundal | Mbilo | 20/12/2021 | River | 06°29'36.6" | 013°15'56.5" |
|  | Tibati | Tibati | Ta'mi | 21/12/2021 | River | 06°27'33.1" | 012°37'16.9" |
|  |  | Tibati | Panyere | 21/12/2021 | Lake | 06°28'09.4" | 012°37'51.4" |
| Faro et Deo | Galim-Tignere | Galim | Mayo Woure | 14/12/2021 | River | 07°04'50.3" | 012°28'08" |
|  |  | Galim | Mayo Njoya | 14/12/2021 | River | 07°06'17.9" | 012°28'26.1" |
|  | Kontcha | Kontcha | Mayo Boudel | 13/12/2021 | Swamp | 07°58'30.3" | 012°14'007" |
|  |  | Kontcha | Okari Laynde Koli | 13/12/2021 | Lake | 07°59'41.0" | 012°14'38.3" |
|  | Mayo baleo | Mayo Baleo | Dokdoure | 12/12/2021 | River | 07°39'19.8" | 012°19'006" |
|  |  | Mayo Baleo | Mayo Baleo | 12/12/2021 | River | 07°38'15.5" | 012°18'51.9" |
|  |  | Alme | Mayo Alti | 13/12/2021 | River | 07°49'47.3" | 012°29'26.9" |
|  |  | Alme | Dilecty | 13/12/2021 | Stream | 07°50'50.2" | 012°26'47.3" |
|  | Tignere | Woulde | Mayo Petel | 11/12/2021 | Stream | 07°26'30.2" | 012°27'46.4" |
| Mayo - Banyo | Bankim | Nyamboya | Mboue | 22/12/2021 | Swamp | 06°17'16.5" | 011°34'37.1" |
|  |  | Bankim | Risière | 22/12/2021 | Stream | 06°04'56.3" | 011°29'04.3" |
|  | Banyo | Banyo | Mayanka | 21/12/2021 | River | 06°44'36.6" | 011°48'12.5" |
|  |  | Banyo | Pendeng | 21/12/2021 | River | 06°44'45.0" | 011°48'57.5" |
|  | Mayo darle | Mayo Darle | Mayo Kodja | 22/12/2021 | River | 06°29'50.0" | 011°33'07.9" |
|  |  | Mayo Darle | Mayo Mbalyara | 22/12/2021 | River | 06°32'12.8" | 011°33'44.7" |
| Mbere | Dir | Dir 1 | Mbigoro | 20/12/2021 | Stream | 06°18'43.2" | 013°35'13.3" |
|  |  | Dir 2 | Barwara | 20/12/2021 | River | 06°19'53.7" | 013°31'51.8" |
|  | Djohong | Djohong | Zakday | 18/12/2021 | River | 06°49'43.0" | 014°41'41.2" |
|  | Meiganga | Meiganga | Zandaba 1 | 20/12/2021 | River | 06°31'35.2" | 014°17'33.4" |
|  |  | Meiganga | Gbakongue | 20/12/2021 | River | 06°29'45.3" | 014°16'31.2" |
|  | Ngaoui | Bafouck | Do'olembe | 18/12/2021 | Stream | 06°39'34.6" | 014°46'43.5" |
|  |  | Bafouck | Koe | 18/12/2021 | River | 06°39'58.2" | 014°47'34.0" |
|  |  | Ngaoui | Gan Mbaie | 18/12/2021 | Stream | 06°45'27.5" | 014°57'04.20" |
| Vina | Belel | Mayo Badji | Mbela | 19/12/2021 | Swamp | 06°52'42.1" | 014°24'14.4" |
|  |  | Belel | Belel | 19/12/2021 | River | 07°03'35.0" | 014°26'08.1" |
|  | Martap | Madira | Madira | 17/12/2021 | Stream | 06°54'00" | 013°03'00" |
|  |  | Bogani | Matong | 17/12/2021 | Stream | 06°53'29" | 013°03'45" |
|  | Mbe | Karna Petel | Mayo Mvôo | 16/12/2021 | River | 07°48'57.2" | 013°34'51.6" |
|  |  | Selou-Semba | Mayo Tchoro | 16/12/2021 | River | 07°48'56.6" | 013°34'51.3" |
|  | Nganha | Berem | Maporo | 16/12/2021 | River | 07°32'34.8" | 013°35'42.5" |
|  |  | Massakbat | Bini | 16/12/2021 | River | 07°32'42.9" | 013°56'46.7" |
|  | Ngaoundere 1 | Beka Hossere | Beka Hossere | 15/12/2021 | Stream | 07°18'28.8" | 013°23'09.3" |
|  |  | Beka Hossere | Mardock | 15/12/2021 | River | 07°20'11.5" | 013°33'46.3" |
|  | Ngaoundere 2 | Djalingo | Djalingo | 15/12/2021 | River | 07°20'30.0" | 013°51'31.0" |
|  |  | Manwi | Manwi | 27/01/2022 | Stream | 07°23'39" | 013°33'40" |
|  | Ngaoundere 3 | Dang | Dang | 27/01/2022 | Lake | 07°25'25" | 013°33'13" |
|  |  | Tchabbal | Tchabbal | 27/01/2022 | Lake | 07°33'24" | 013°34'21" |
|  | Nyambaka | Nyambaka | Mambaka | 19/12/2021 | River | 06°54'06.0" | 014°07'52.3" |
|  |  | Nyambaka | Abattoir | 19/12/2021 | Stream | 06°53'16.0" | 014°05'31.3" |
