# Supplementary figures and images for: Mapping and phylogeny of Biomphalaria snail in the Adamawa Region of Cameroon: A step towards vector control and schistosomiasis elimination
Source: PLoS Negl Trop Dis. 2025 Jun 27;19(6):e0013265. doi: 10.1371/journal.pntd.0013265 (PMC12244607; doi:10.1371/journal.pntd.0013265)

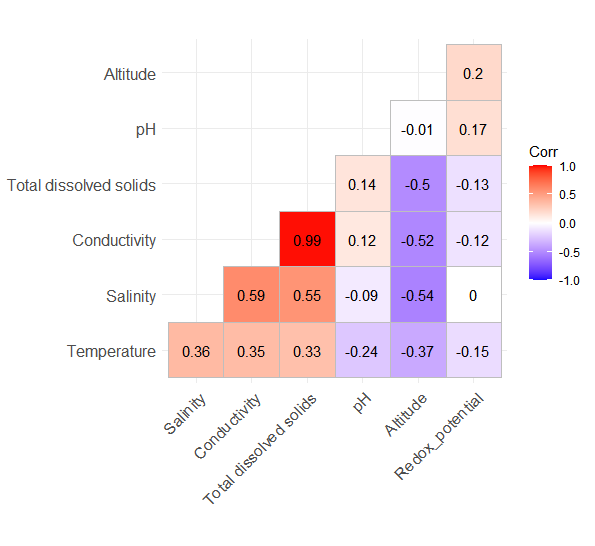

Supplement: S1 Fig — (TIF) [file pntd.0013265.s003.tif]
